# Notational usage modulates attention networks in binumerates

**DOI:** 10.3389/fnhum.2014.00326

**Published:** 2014-05-28

**Authors:** Atesh Koul, Vaibhav Tyagi, Nandini C. Singh

**Affiliations:** National Brain Research Centre, Deemed UniversityGurgaon, India

**Keywords:** binumerates, Nagari, fMRI, numeracy, notation, attention

## Abstract

Multicultural environments require learning multiple number notations wherein some are encountered more frequently than others. This leads to differences in exposure and consequently differences in usage between notations. We find that differential notational usage imposes a significant neurocognitive load on number processing. Despite simultaneous acquisition, twenty four adult binumerates, familiar with two positional writing systems namely Hindu *Nagari* digits and Hindu *Arabic* digits, reported significantly lower preference and usage for *Nagari* as compared to *Arabic*. Twenty-four participants showed significantly increased reaction times and reduced accuracy while performing magnitude comparison tasks in Nagari with respect to Arabic. Functional magnetic resonance imaging revealed that processing Nagari elicited significantly greater activity in number processing and attention networks. A direct subtraction of networks for Nagari and Arabic notations revealed a neural circuit comprising of bilateral Intra-parietal Sulcus (IPS), Inferior and Mid Frontal Gyri, Fusiform Gyrus and the Anterior Cingulate Cortex (FDR *p* < 0.005). Additionally, whole brain correlation analysis showed that activity in the left inferior parietal region was modulated by task performance in Nagari. We attribute the increased activation in Nagari to increased task difficulty due to infrequent exposure and usage. Our results reiterate the role of left IPS in modulating performance in numeric tasks and highlight the role of the attention network for monitoring symbolic notation mode in binumerates.

## Introduction

Numbers are an integral part of life and are represented as distinct symbolic notations across varying cultural environments. Investigations of neural mechanisms underlying number processing have established a network of brain regions comprising bilateral Intra-parietal sulcus (IPS) (Piazza et al., 2007; Holloway et al., 2010), fusiform and prefrontal cortex in numerical cognition (Arsalidou and Taylor, 2011; Emerson and Cantlon, 2012). Specifically, IPS has been suggested to be an amodal, notation independent substrate for mathematical abilities (Chochon et al., 1999; Naccache and Dehaene, 2001; Eger et al., 2003; Piazza et al., 2004) while frontal regions like the inferior and middle frontal gyrus (IFG and MFG) have been suggested to be involved in processes like working memory, sequencing, controlled retrieval and decision making during arithmetic tasks (Gruber et al., 2001; Bor and Owen, 2007; Pinel and Dehaene, 2010). More recently, electrophysiology studies in primates and neuro-stimulation research have also implicated areas in the pre-frontal cortex with spatial representation of numbers (Rusconi et al., 2011), in creating associations between numerical symbols and numerical representations (Diester and Nieder, 2007) as well as in the encoding of mathematical rules (Bongard and Nieder, 2010). Thus, a substantial body of research over the last two decades has demonstrated the universality of activation of the IPS, pre-frontal regions and ventral visual areas in tasks involving number processing.

Despite universality of the core number processing network, comparisons of task performance in distinct symbolic number notations have suggested differences in neural processing across notations. For instance, number recognition tasks in Japanese-English bilinguals involving Kana, Kanji and Arabic (Ar) numerals, showed differential activations while processing the three notations (Coderre et al., 2009). Specifically, additional activation in the posterior cingulate was observed while processing syllabic and phonetic Kana numbers when compared to logographic representations of Kanji and Ar notations. Similarly, another study with Roman and Ar numerals showed reduced accuracy and increased reaction times with less familiar Roman numerals when compared to well-rehearsed and automated Ar notation during mental arithmetic tasks (Wu et al., 2009). On the other hand, the study by Wu et al. (2009) found increased activations in the pre-frontal areas while processing Roman numerals as compared to Ar.

Indeed, in multicultural environments where multiple notations are used, some notations are encountered more often than others, which can lead to differences in performance and neural activation (Marsh and Maki, 1976; Perani et al., 1998; Lin et al., 2011). Past literature thus points toward two possibilities. Firstly, infrequently used, less familiar notations can activate additional cortical regions like posterior cingulate as compared to more frequently encountered notations like (Coderre et al., 2009). Alternatively less familiar notations can activate same regions of the brain as activated by familiar notations but with higher levels of activation (Wu et al., 2009).

Japanese uses both syllabic (kana) and logographic (kanji) writing systems. While Japanese kana allows a direct phonetic reading, as each symbol represents a syllable, and is analogous to digit words (i.e., “three”), Japanese kanji uses Chinese characters with modified pronunciations, and like Arabic numbers require the pronunciation of each individual character to be memorized (i.e., there are no phonetic clues in the symbol “4” that indicate its pronunciation, “four”). Further, both Ar and kanji are used with near-equal frequency and do not differ in familiarity or usage (Coderre et al., 2009). Thus, despite differences in the surface features of Ar and Kanji, since they both use similar strategies, and are used equally frequently, no differences in task performance or neural representation were observed. Kana, on the other hand, has been suggested to use a different strategy for number identification and is also infrequently used. As a result, it is difficult to ascertain if the increased reaction time and additional activation in the posterior cingulate seen while performing number identification in kana was due to differences in number processing strategy or usage.

Similarly in the study comparing Ar and Roman numerals by Wu et al. (2009) only increased activation was seen in the prefrontal cortex for Roman as compared to Ar, which were attributed to increased task difficulty in less automated, less familiar Roman numerals. However, no additional regions were activated while processing Roman numerals. Additionally, Roman number system does not follow a positional number-writing system but uses subtractive or additive principles (Holender and Peereman, 1987). For instance, placing any smaller number in front of any larger number, like I in front of the V, indicates subtraction and placing I after the V means addition and so on. On the other hand, Arabic number system is a positional number-writing system where each logographic symbol represents a quantity. Thus, processing of Roman numerals does not necessarily rely only on memorization like the Ar numerals. It is possible that in addition to infrequent usage, differences in strategy while processing Roman as compared to Ar numbers also contributed to the prefrontal activation seen in Wu et al. (2009).

To summarize, while both studies reported increased reaction time and reduced accuracy for tasks being performed in the less familiar, infrequently used number notation, the brain regions reported were different. Secondly, inherent differences in the strategies of encoding/representing the less familiar notation might have confounded the interpretation of the results.

**Figure 1:**
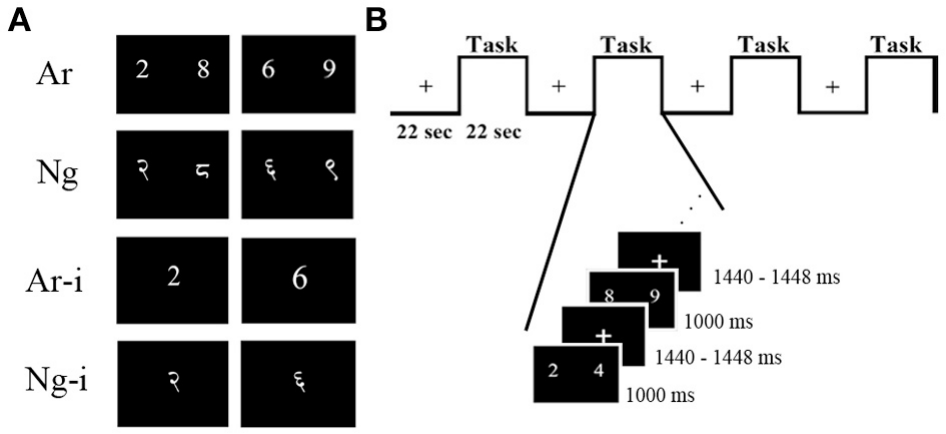
**Examples of stimuli and task design**. **(A)** Two symbolic notations were used as stimuli for number comparison (Ar, Ng) and number identification (Ar-i, Ng-i) tasks. Participants chose which of the two numbers was greater in magnitude in number comparison task and named the presented number in number identification task, **(B)** details of experimental design and timing for one comparison run. Each run had four task blocks alternating with four rest blocks.

The current study was undertaken to resolve the issues discussed above. We investigated effects of usage and task difficulty in Nagari-Arabic (Ng-Ar) binumerates, individuals who learn and use more than one number system as part of their academic curriculum. Socio-academic patterns in India lend themselves ideally to investigate effects of differential usage of notations. Ng-Ar binumerates in India simultaneously acquire two distinct number notations, namely Hindu-Arabic digits (Ar) and Hindu-Nagari digits (Ng) (Figure 1A). Nevertheless, due to a shift in academic instruction towards Ar as well as reduced usage of Ng in social context, the exposure to Ar increases. This gives rise to a disproportionate level of familiarity and usage between notations. Thus, while Ar-Ng binumerates are familiar with both notations they rely on Ar notations for daily operations and transactions, which results in differential familiarity and usage of Ar compared to Ng. We exploit this natural environment to investigate the effect of notational usage on task performance and neural activation patterns. Additionally since Ar numerals are derived from Ng numerals, both Ng and Ar numerals are positional number-writing systems, logographic in nature, have similar surface features (Smith and Karpinski, 2013) and likely use similar strategies for number processing. These features permit the specific investigation of usage on the task performance and neural activation.

A second hypothesis of interest in this study is that related to conflict monitoring and control. The anterior cingulate cortex (ACC) is an important component in the neural circuit mediating cognitive control and closely tied to monitoring conflicting information (Carter et al., 1999; Botvinick et al., 2004). The requirement of such cognitive control is obvious in bilinguals who must select and monitor language for interaction and discourse (Abutalebi et al., 2011). As a consequence when required to perform tasks in a specific language, bilinguals need to select and monitor the target language while inhibiting response in the other language. We hypothesize similar conflict monitoring mechanisms in binumerates, wherein the knowledge of two symbolic notations also necessitate successful selection of target symbolic notation mode and inhibition of the other. We postulate therefore the recruitment of possibly the ACC along with other neural substrates that may be involved in conflict monitoring during task performance.

The objectives of the current study were—(i) to investigate the effect of differential usage of two symbolic number notations on mathematical task performance, (ii) to delineate the neural basis of increased mathematical task difficulty while using less frequently used notation and (iii) to identify neural correlates that modulate individual differences in task performance. Since Ng notation is less familiar and is less frequently used, we hypothesized that Ng would be less preferred than Ar and that number processing tasks in Ng would be more difficult to process as compared to those in Ar. At the behavioral level, we anticipated increased reaction times while processing Ng, while at the neural level, we hypothesized either increased activations in the fronto-parietal network and/or recruitment of additional areas while processing Ng.

## Materials and methods

### Participant selection and assessment

Twenty four right handed biscriptal binumerates (9 females, mean age = 23.64 years, *SD* = 3.18) from the National Capital Region Delhi, India, familiar with two number notations (Ar and Ng), participated in the study. All participants were healthy, had normal or corrected-to-normal vision and had no neurological or psychiatric disorders. Handedness was tested using a modified Edinburgh Handedness Questionnaire (Oldfield, 1971). The Human Ethics Committee of the Centre approved all behavioral and neuroimaging protocols. The participant pool included bilingual individuals who were simultaneously exposed to only two languages—Hindi (Hi) and English (En). They were recruited using advertised notices in different institutes of the National Capital Region of Delhi, in the northern part of India.

The native language of all participants was Hindi and they had received formal mathematical education for a minimum of 10 years as reported in a language and mathematical ability questionnaire. A two-part questionnaire to evaluate participants' mathematical ability and preference across notations was designed. In part A, participants' familiarity with Ar and Ng was determined using self-reported subjective ratings, while part B assessed participants' numerical abilities through an objective test. In part A, participants rated their preferences for mental (on a scale of 1–5) and written mathematical operations (on a scale of 1–3) for the two notations. Questions on mental operations comprised of eight different arithmetic concepts including addition, subtraction, multiplication, division, percentage calculation and graphical representation. Participants' preference for written mathematical operations was probed using five questions on everyday activities like filling out a bank form, writing a phone number, listing shopping items and adding bills. Part B comprised of a series of 15 questions that included single, double and triple digit additions and subtractions in Ar and Ng notations. Detailed demographic information for 19 participants is provided in Table 1. For reasons beyond our control five participants were unable to complete the questionnaire designed to elicit demographic information. All 24 participants participated and completed the neuroimaging study.

**Table 1:** **Demographics, preference and performance of the participants as measured by a language and mathematics questionnaire**.

**DEMOGRAPHICS (*n* = 19)**		
Mean age (years)		22.95 (0.72)
Formal mathematical education (years)		13 (0.63)
**LANGUAGE PREFERENCE (*n* = 19)**		
	**En**	**Hi**
Self-reported proficiency (1–5)	4.03 (0.15)	4.71 (0.09)
**MATHEMATICAL TEST PROFICIENCY (*n* = 19)**		
	**Ar**	**Ng**
Objective test scores (%)	88.77 (2.45)	81.05 (3.19)
**PREFERENCE FOR MATHEMATICAL NOTATIONS (*n* = 19)**		
	**Ar**	**Ng**
Mental operations (1–5)	4.72 (0.11)	2.43 (0.21)
Written operations (1–3)	2.99 (0.01)	1.20 (0.11)

*Participants reported their preference of usage across languages and mathematical notations. Their performance across notations was measured using an objective test comprising of one, two and three digit mathematical operations*.

### Experimental task and timing parameters

Participants performed two experiments: number comparison and digit identification in Ar and Ng. Symbolic number comparison tasks were used since they specifically involve magnitude representation and are devoid of any complex arithmetic processing (Zorzi et al., 2011). During number identification tasks, participants identified and named a number presented on the screen subvocally.

During number comparison tasks, two numbers were presented simultaneously and participants were asked to indicate the larger number (in magnitude) by a button press as quickly and as accurately as possible. During rest conditions, participants fixated on a cross appearing at the center of the screen. All participants were given detailed instructions about the experiment in both Hindi and English before entering the scanner. They also completed a practice run before the main task in the scanner. The two sets of tasks were presented on a computer screen projected onto a mirror assembly mounted on the MRI head coil.

Participants performed Ar and Ng number comparison tasks in two runs, separated by a perceptual task. This was followed by the digit identification tasks for Ar and Ng in two separate runs. Each run was divided into eight blocks consisting of alternating rest and task blocks. Each block was 22 s long and in turn comprised of nine trials in case of number comparison and 12 trials in case of number identification task. Each trial consisted of a task screen followed by a jittered inter-trial-interval (Figure 1B). All runs of this block design experiment were created and presented using E-Prime v1.0 (Psychological Software Tools) presentation software. In number comparison tasks, participants were instructed to judge the two numbers presented on the screen and provide a button press response toward the side which had the larger number using either their index finger or the middle finger of the same hand. In order to control for SNARC effect (Dehaene, 1993), the order of hand usage was counterbalanced over subjects, that is, half of the participants used their left hand fingers to indicate their response while other half used their right hand.

### Stimuli

Picture stimuli for all tasks were constructed using Adobe® Photoshop® (Adobe Systems Incorporated, San Jose, CA, USA). Ar stimuli were displayed in Times New Roman font while Ng stimuli were typed in Shangrila Numeric Regular font (Figure 1A). All symbolic number stimuli were matched for size (70 ± 2 pixels high). Numbers from 1 to 9 except 5 were used for all tasks. The same number pairs were used for Nagari notation. All stimuli were presented equidistant from a central fixation point in black on a white 1024 × 768 resolution screen.

### fMRI data acquisition

T1 weighted structural and T2^*^ weighted functional magnetic resonance images were collected in a whole body 3T Philips Achieva scanner using an 8-channel Philips Sense head coil. High resolution 3D T1-weighted images consisting of 150 slices were acquired (FOV = 250 × 230 mm, matrix size = 252 × 205 reconstructed to 256 × 256). Gradient echo-planar imaging T2^*^-sequence sensitive to blood oxygenation level-dependent (BOLD) contrast was used to acquire functional images. Functional images were collected in a descending order (*TR* = 2.2 s, *TE* = 30 ms, flip angle = 90°, FOV = 230 × 230 mm, voxel size = 3.5 × 3.5 × 3.5 mm, matrix size of 64 × 64 reconstructed to 128 × 128). Forty five transverse slices covering the entire brain were collected (slice thickness 3.5 mm, gap = 0 mm). For each run of comparison and identification tasks, 80 volumes were acquired with a total of 320 volumes per participant. The first two scans of each run were discarded to allow for equilibration of magnetization.

### Imaging data analysis

Data analysis was performed using SPM8 (v 4290) (Wellcome Trust Centre for Neuroimaging, University College London; http://www.fil.ion.ucl.ac.uk/spm). The EPI images were first realigned to the mean image and then co-registered with respective high resolution T1 images. Data from participants whose within-run and between-run translation head motion was more than 3 mm and rotational head motion more than 2° were excluded from the analysis. The T1 images were segmented and normalization parameters to MNI space were calculated. The functional images were then normalized to MNI space using the parameters from segmentation. The images were smoothed using a Gaussian smoothing kernel (FWHM = 8 mm). A whole brain GLM analysis was carried out to find regions of activation in single subjects using a canonical HRF basis function and a high pass filter of 128 s. Subsequently, a random effects analysis (one sample *t*-tests) was carried out to obtain common regions activated in the group (Ar>Rest, Ng>Rest). After second level analysis of data, activation maps from comparison tasks were inclusively masked by their corresponding identification tasks i.e., Ar comparison task masked by Ar identification task and Ng comparison by Ng identification task. This was done in order to avoid general activations related to action. All the comparison results thus, include only the regions specific to number processing rather than to response selection. Further, a paired *t*-test (Ng-Rest>Ar-Rest) was used to investigate effect of difficulty on neural activation.

All imaging results were corrected for multiple comparisons using FDR correction (Genovese et al., 2002; Poldrack et al., 2011).

A whole brain regression analysis was performed to examine the correlation between reaction time and activation in comparison tasks. First Eigen variates from a spherical ROI of radius 8 mm, centered at the peak voxel of the cluster were used for the correlation.

## Results

### Questionnaire results

Participants' subjective ratings on questions in mental and written mathematical operations were averaged for each participant to obtain mean subjective ratings across all notations. These mean subjective ratings for all participants were then averaged to obtain a mean preference rating for both notations (Boone and Boone, 2012).

#### Preference ratings for mental mathematical operations

A paired *t*-test between Ar and Ng notation for preference in mental mathematical operations showed a significant difference (*p* < 0.001). Ar notation was significantly more preferred than Ng notation (Table 1).

#### Preference ratings for written mathematical operations

A similar *t*-test for preference in written mathematical operations revealed a significant difference (*p* < 0.001). Ar was significantly more preferred than Ng for written mathematical operations (Table 1).

#### Performance on objective test

Mean accuracy of participants was analyzed using a paired *t*-test. Participants were significantly accurate on Ar as compared to Ng test questions (*p* < 0.005) (Table 1).

### Behavioral results

**Figure 2:**
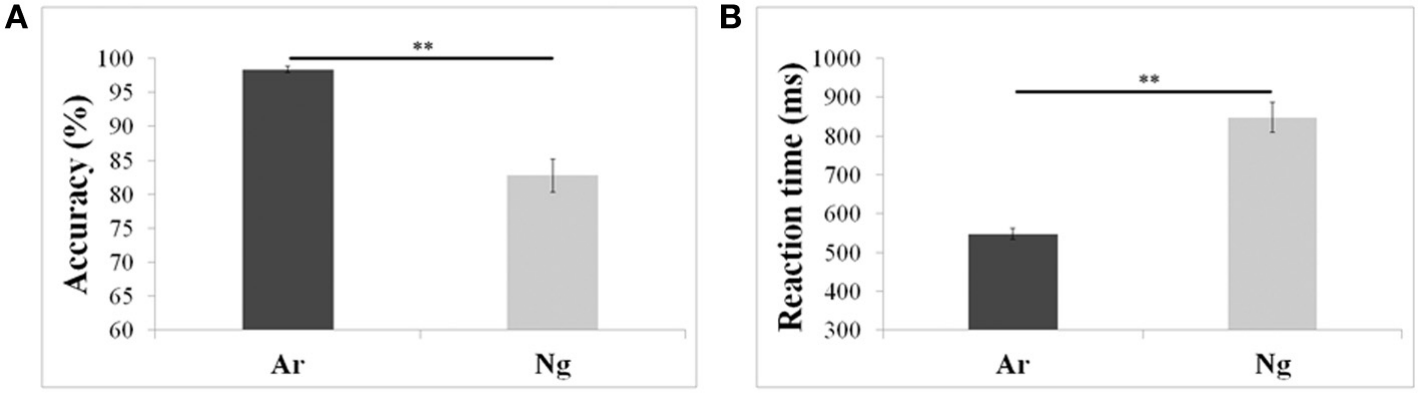
**Performance measures of participants in Ar and Ng tasks**. **(A)** Percent accuracies and **(B)** reaction time in milliseconds for Arabic and Nagari number comparison tasks. ^**^indicates significance at *p* < 0.001. Error bars represent standard error of the mean.

Mean reaction times and accuracies of 24 participants who participated in the neuroimaging experiments were analyzed using a paired *t*-test. Participants were more accurate and faster (*p* < 0.001) for Ar compared to Ng task (Figure 2; Table 2).

**Table 2:** **Behavioral performance of the participants**.

	**Ar**	**Ng**
Accuracy	0.98 (0.004)	0.83 (0.024)
Reaction time (in ms)	547.74 (13.98)	847.78 (38.57)

*Figures in parentheses indicate standard error of mean (s.e.m)*.

### Imaging results

#### Digit identification tasks

**Figure 3:**
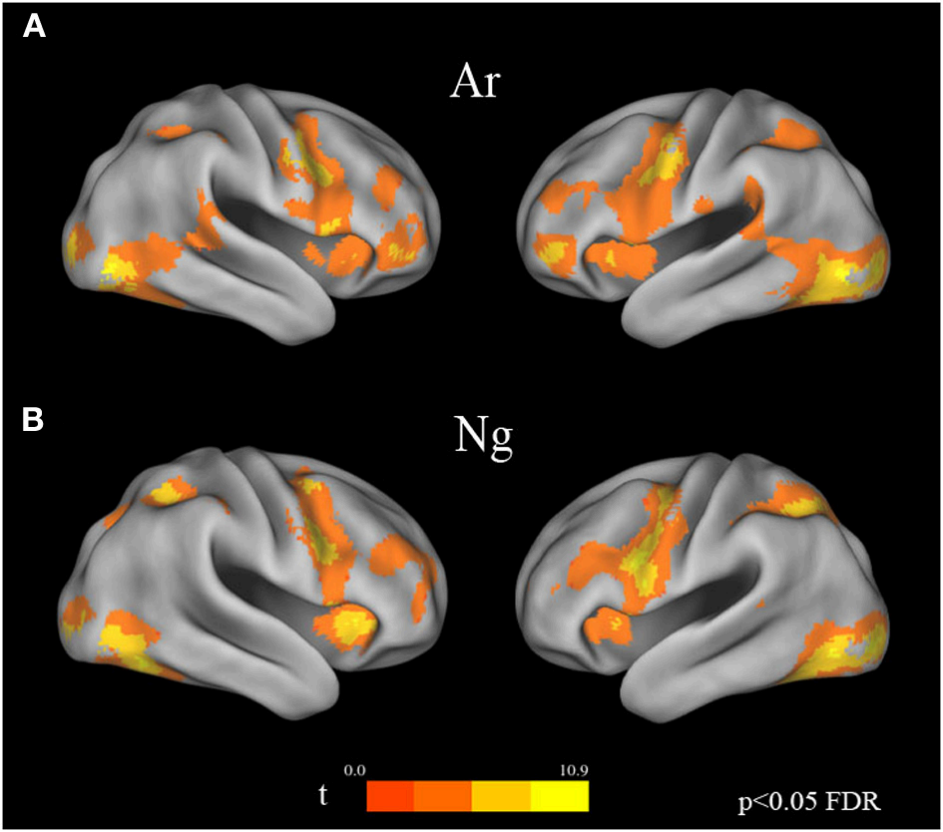
**Brain activation maps for Ar and Ng number identification tasks**. Regions of activation in the brain for **(A)** Ar and **(B)** Ng tasks are shown as color coded brain maps (FDR *p* < 0.05).

Voxel wise random effects analysis on Ar and Ng Identification tasks revealed a bilateral activation of IPS, IFG and fusiform gyrus at a corrected threshold (*p* < 0.05 FDR) (Figure 3). Other regions of activation included bilateral MFG, supplementary motor area (SMA)/cingulate gyrus. No differences between activation patterns when identifying digits in Ar and Ng were found (either in Ar>Ng or Ng>Ar contrast) as revealed by a paired *t*-test (*p* < 0.05 FDR).

#### Number comparison tasks

**Figure 4:**
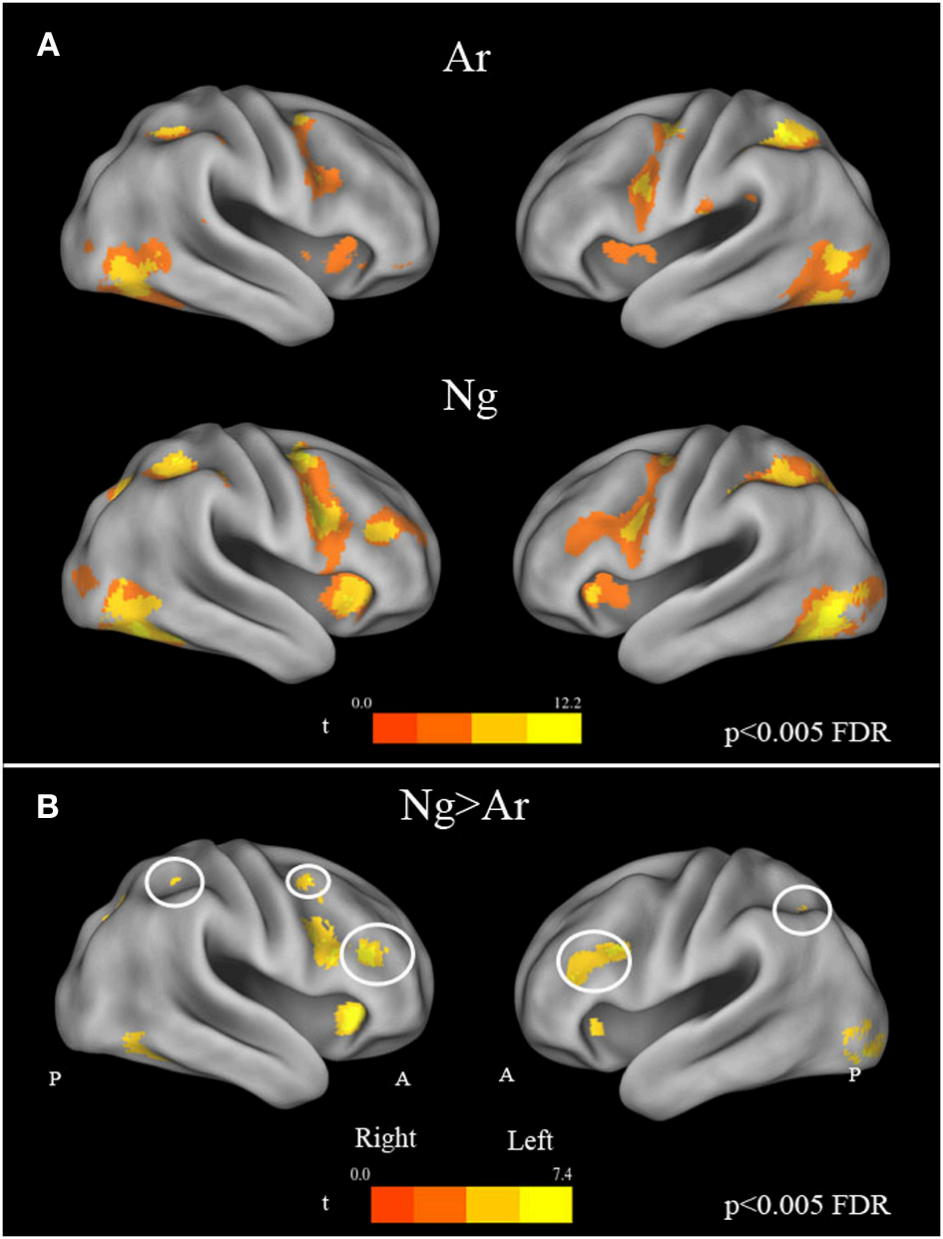
**(A)** Brain activation maps for Ar and Ng number comparison tasks. All maps are masked by their respective number identification masks. Statistical maps were corrected for multiple comparisons (FDR correction at *p* < 0.005). **(B)** Effect of task difficulty. Brain regions activated in Nagari as compared to Arabic contrast (FDR, *p* < 0.005) masked by Ar and Ng identification mask. A reverse Arabic >Nagari contrast, did not reveal activations in any brain area. Regions of the Fronto-parietal network with increased activation are outlined in white.

A whole brain random effects analysis (one sample *t*-test) was performed to find regions activated by comparison task across notations. This was followed by an inclusive masking by activation maps from identification tasks for each notation in order to control for the areas activated by cognitive factors other than number processing such as button press. Both tasks showed activations in bilateral parietal and frontal regions as compared to baseline. Specifically, bilateral activations in IPS, fusiform gyrus, precentral gyrus, IFG, SMA/cingulate gyrus and cerebellum were found (Figure 4A) (see Table 3). In order to control for differences in activations due to performance, behavioral measures were regressed out for each task in the analysis. However, no significant differences in the activation pattern were seen (Supplementary Figure 1).

**Table 3:** **Peak coordinates from significant regions of activation in Ar and Ng comparison tasks**.

**Anatomical area**	**Left hemisphere**				**Right hemisphere**			
	***t*-scores**	**Peak MNI coordinates**			***t*-scores**	**Peak MNI coordinates**		
		***x***	***y***	***z***		***x***	***y***	***Z***
**HINDU ARABIC DIGITS**								
Intraparietal sulcus	9.38	−28	−56	53	5.95	30	−54	48
Fusiform gyrus	7.46	−38	−76	−18	5.8	44	−72	−18
Mid frontal gyrus					6.55	32	46	18
SMA/cingulate gyrus	6.13	−4	4	48	6.15	4	14	48
Inferior frontal gyrus	7.97	−56	11	25	5.57	52	8	22
Precentral gyrus	7.48	−40	−10	54	5.94	50	10	30
Cerebellum	6.25	−34	−54	−26	6.82	32	−58	−54
**HINDU NAGARI DIGITS**								
Intraparietal sulcus	7.73	−30	−54	50	6.56	32	−52	44
Fusiform/inferior occipital gyrus	10.87	−48	−74	−4	6.94	40	−76	−10
Mid frontal gyrus	4.63	−36	42	26	8.15	38	2	56
SMA/cingulate gyrus	9.12	2	14	46	7.16	14	6	50
Inferior frontal gyrus	7.85	−42	6	22	4.55	55	20	16
Precentral gyrus	9.16	−36	−6	52	10.77	46	4	32
Cerebellum	11.80	−45	−62	−28	11.15	48	−60	−26

*Activation peaks that passed a voxel-wise whole brain FDR threshold p < 0.005 are reported*.

#### Effect of task difficulty on neural activation

To investigate the effect of task difficulty on brain activations, we performed a paired *t*-test on Ar and Ng task. The contrast for Ng>Ar during number magnitude comparison revealed a group of cortical areas which include bilateral IPS, IFG and MFG and ACC (*p* < 0.005 FDR) (Figure 4B) (see Table 4). A reverse contrast of Ar>Ng did not show any regions of activation (*p* < 0.005 FDR). Furthermore, an analysis of unmasked Ng>Ar contrast (without masking with the digit identification task) revealed additional regions of activation in the inferior temporal and prefrontal regions (Supplementary Table 1, Supplementary Figure 2).

**Table 4:** **Brain areas significantly activated in Ng>Ar contrast**.

**Anatomical area**	**Left hemisphere**				**Right hemisphere**			
	***t*-scores**	**Peak MNI coordinates**			***t*-scores**	**Peak MNI coordinates**		
		***x***	***y***	***z***		***x***	***y***	***z***
Intraparietal sulcus	4.58	−29	−65	43	3.63	38	−52	52
Fusiform/inferior occipital gyrus	5.51	−36	−88	−8	3.70	35	−89	−5
Mid frontal gyrus	3.69	−26	2	54	4.94	35	8	56
SMA/cingulate gyrus	7.42	−2	20	44	4.02	11	12	50
Inferior frontal gyrus	6.81	−40	24	22	6.21	46	28	18
Precentral gyrus	3.52	−46	−2	40	5.64	50	10	34
Cerebellum	5.38	−48	−60	−26	5.28	36	−52	−38

*Activation peaks that passed a voxel-wise whole brain FDR threshold p < 0.005 are reported*.

#### Brain behavior correlation

**Figure 5:**
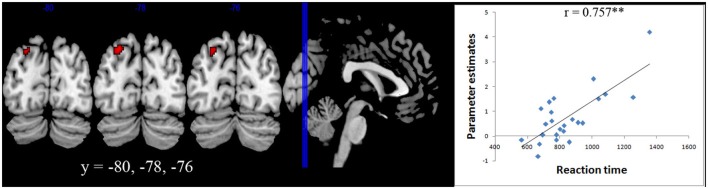
**Brain-behavior correlation**. Whole brain regression analysis with reaction time for Nagari task showed a significant correlation (*p* < 0.05 FDR) with activation in Left IPS centered at (−24 −78 44).

A whole brain regression analysis with both reaction time and accuracy with brain activity during number comparisons were carried out for both Ar and Ng. A significant correlation with activation in Left IPS (centered at −24 −78 44) was found only for the Ng task (*p* < 0.05 FDR) (Figure 5).

## Discussion

The current study assessed the effect of notation usage on task performance in Ng-Ar binumerates performing numerical magnitude comparison in two symbolic notations namely Nagari and Arabic. Behavioral data showed that participants were significantly slower and less accurate while performing magnitude comparisons in Ng as compared to Ar. In terms of brain activity, both Ar and Ng number comparisons activated a network of brain regions comprising of IPS, IFG, SMA/cingulate and fusiform gyrus bilaterally. Significantly higher brain activity for processing Ng as compared to Ar was found in cortical areas associated with attention and cognitive control (Figure 4). The mathematical ability questionnaire ratings for Ar and Ng notations clearly indicated differences in usage for Ar and Ng notations. As indicated in Table 1, for both mental and written mathematical operations, participants' reports indicated increased Ar usage over Ng. Not surprisingly, participants also reflected this familiarity of notation usage in their behavioral performance wherein they performed significantly slower and less accurately on number magnitude comparison tasks in Ng when compared to Ar (Figure 2). Previous studies using symbolic notations have shown effects of notation familiarity on automaticity and task performance. As reported by Wu et al. (2009), participants showed reduced automaticity and increased reaction times while performing addition tasks in Roman as compared Ar symbols. Interestingly, despite the fact that Ng was acquired along with Ar and did not show differences in the symbol identification task, the regular usage of Ar symbols clearly impacted task performance. We discuss below the neural consequences of processing two symbolic notations in binumerate populations.

Our binumerate participants showed significant effects of infrequent usage of Ng on brain activity. While the neural networks for numerical magnitude comparisons for both Ng and Ar were similar, significantly greater brain activity was seen for Ng as compared to Ar. We found bilateral activations in the IPS, IFG and fusiform gyrus for magnitude comparison tasks in both Ng and Ar number notations (Figure 4). This is in agreement with past studies on number comparison tasks that have demonstrated involvement of bilateral IPS and IFG in Ar notation (Chochon et al., 1999; Pesenti et al., 2000; Holloway et al., 2010). While activations in IPS have been suggested to be magnitude specific (Dehaene et al., 2003; Piazza and Izard, 2009; Dormal et al., 2010) those in frontal regions have been attributed to working memory processing (Bor and Owen, 2007) as well as spatial representation of numbers (Rusconi et al., 2011). Our findings are also consistent with Dehaene's triple code model where the fusiform gyrus is proposed to serve as visual number area, left IFG as verbal system and IPS as the seat of numerical magnitude representation (Dehaene, 1992; Dehaene and Cohen, 1995). The findings of our study also confirmed IPS activation across tasks irrespective of stimulus notation. The current study is also the first to report neural circuits responsible for processing Ng.

A direct subtraction Ng compared with Ar revealed significant activity in a network of regions comprising bilateral IPS, IFG, MFG, fusiform gyrus and the ACC. Importantly, no regions were significantly more active for Ar compared to Ng. The increased activation while processing Ng may be attributed to two possibilities (1) differences in surface features of Ng and Ar numerals or (2) infrequent usage of Ng symbols resulting in increased cognitive load during the Ng task. Our results of number identification of Ng and Ar in Figure 3 indicate no differences in neural representation. Additionally since Ng and Ar are both positional (or abstract place-value) number writing systems that do not differ in strategies for performing magnitude comparisons, differences in neural activity between Ng and Ar investigated in the current study may be attributed solely to the effect of notation usage on neural processing.

The results from Ng>Ar contrast in the current work were masked by corresponding digit identification tasks which might obscure additional activations not related to perception. An analysis of unmasked Ng>Ar contrast was also performed which showed additional regions of prefrontal cortex—inferior and middle frontal gyri as well as inferior temporal gyrus (Supplementary Figure 2).

The increased activity in fronto-parietal network during the Ng task (in both masked and unmasked contrasts) suggests an increased attentional demand. Increased activation in IFG and MFG have previously been implicated in working memory load (Ischebeck et al., 2006) and general attention (Curtis and D'Esposito, 2003; Owen et al., 2005). For instance, studies on arithmetic learning in adults by (Delazer et al., 2003) report greater attentional demands and thus stronger activation of IFG and IPS for non-automated and complex calculations. On the other hand, increase in numerical proficiency with arithmetic calculations like addition, multiplication and subtraction have been associated with decrease in activation levels of frontal and parietal regions (Ischebeck et al., 2006, 2007; Zamarian et al., 2009). Specifically, a decrease in activation of frontal and parietal regions was observed while participants were being trained on novel addition, multiplication and subtraction problems. A reduced working memory load on prefrontal lobes (in the form of reduced prefrontal activations) was observed while the participants were being trained. We therefore attribute the increased activation in MFG and IFG while processing Ng to the increased attentional demand that arises due to increased working memory load experienced while processing infrequently used Ng.

The greater recruitment of ACC and SMA while processing Ng numerals may be attributed to cognitive control, necessary in populations with knowledge of multiple symbolic notations. A host of studies on language mode in bilinguals have shown that these areas are associated with cognitive control. For instance, Chinese–English bilinguals showed increased activity in the ACC and pre-SMA when they named pictures in English as compared to Chinese (Guo et al., 2011). Along similar lines, a recent study by Rao et al. also showed increased activity in the anterior cingulate and pre-SMA when Hindi-English bilinguals identified abstract and concrete nouns in less frequently encountered Romanized transliteration (Romanized Hindi) as compared to Hindi (L1) and English (Rao et al., 2013).The ACC and pre-SMA have now been consistently reported as components of a cognitive control network, responsible for monitoring language mode and for controlling interference from the non-target language(s) in bilinguals (Abutalebi and Green, 2007; Abutalebi, 2008). We attribute a similar view in binumerates and postulate that similar to bilinguals, in binumerates too; activity in the anterior cingulate and pre-SMA is required to maintain a specific symbolic notation and additional activity for the less frequently used notation is a signature of additional neurocognitive effort.

The difference in performance of participants in the Ng task also allowed us to investigate neural correlates modulated by task performance. A whole brain correlation with reaction time in Ng showed a significant positive correlation in the left intra-parietal cortex (Figure 5). Our results therefore suggest that individual differences in activity in the left parietal cortex during numerical magnitude processing in Ng may be related to variability in task performance. Our findings are similar to those reported in developmental studies wherein individual differences in neural processing of numerical magnitude were positively correlated with activity in the left IPS (Bugden et al., 2012). However in that study, individual differences in numerical magnitude were correlated with differences in math fluency scores. In our study, since individual differences in task performance are correlated with differences in usage, we suggest that differences in the activity of the left intraparietal cortex also provide information about notation usage and automaticity.

In summary, our study shows that symbolic notation usage imposes significant cognitive effects on numerical magnitude processing that are reflected at the behavioral and neural level. Behaviorally this is manifested in terms of increased reaction time and reduced accuracy during magnitude comparison tasks and neurally is evident in increased activity of the attentional network comprising of a bilateral frontal-parietal network and the fusiform gyrus. Additionally, it also highlights the fact that similar to bilinguals, even binumerate populations exposed to processing two symbolic notation systems are required to also activate regions involved in conflict monitoring namely the ACC and SMA in order to monitor symbolic notation mode during task performance. Finally, this study separates the effects of familiarity with a notational system from those of processing strategy arising from the nature of the system itself.

### Conflict of interest statement

The authors declare that the research was conducted in the absence of any commercial or financial relationships that could be construed as a potential conflict of interest.

## Supplementary material

Supplementary Table 1 **Brain areas significantly activated in unmasked Ng>Ar contrast (without masking with digit identification task) in addition to the areas found in masked contrast**.

Supplementary Figure 1 **Brain activation maps for Ar and Ng number comparison after regressing out behavioral measures for each task**.

Supplementary Figure 2 **Brain regions activated in unmasked Ng>Ar contrast (without masking with digit identification task)**.
