# Supplementary material for: Notational usage modulates attention networks in binumerates
Source: Front Hum Neurosci. 2014 May 28;8:326. doi: 10.3389/fnhum.2014.00326 (PMC4035602; doi:10.3389/fnhum.2014.00326)

Supplementary table 1: Brain areas significantly activated in unmasked Ng>Ar contrast (without masking with digit identification task) in addition to the areas found in masked contrast

**Anatomical area**	**Left hemisphere**				**Right hemisphere**			
	**t-scores**	**Peak MNI coordinates**			**t-scores**	**Peak MNI coordinates**		
		**x**	**y**	**Z**		**x**	**y**	**z**
Inferior Temporal gyrus					6.99	48	-46	-8
Inferior Frontal gyrus	5.97	-37	27	21	6.70	50	38	12
Cuneus					6.17	14	-74	42
Precuneus	4.88	-4	-73	49				
Mid Frontal gyrus	5.12	-44	52	-2	5.60	26	15	41
Calcarine sulcus	5.66	-10	-84	8				
Mid Occipital gyrus					4.90	36	-66	36

Supplementary figure 1: Brain activation maps for Ar and Ng number comparison after regressing out behavioral measures for each task.

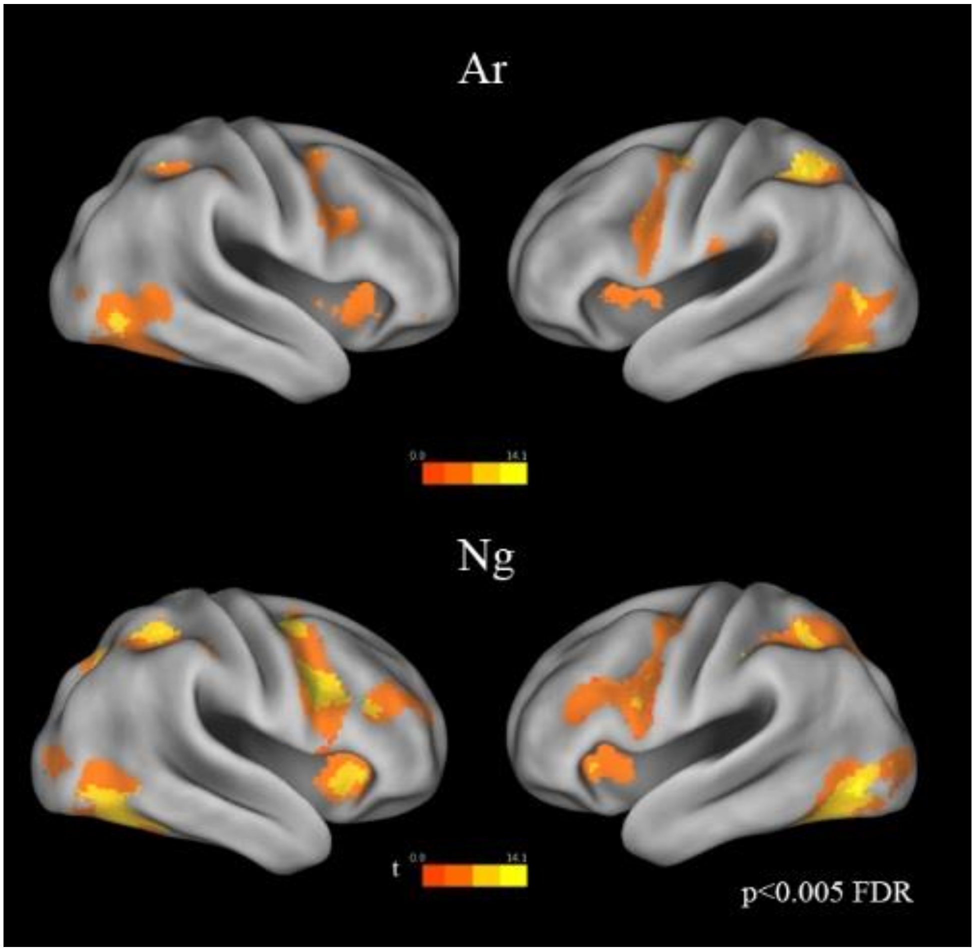

Supplementary figure 2: Brain regions activated in unmasked Ng>Ar contrast (without masking with digit identification task)

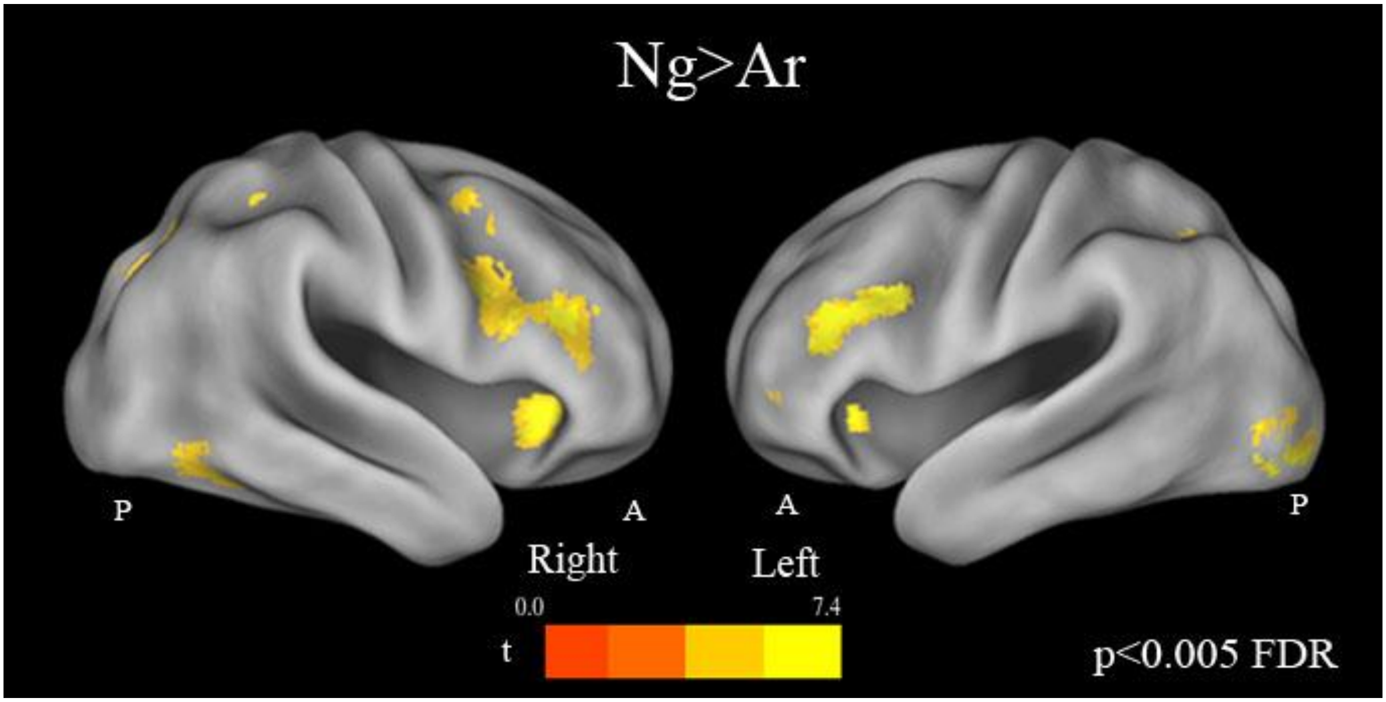

Supplement: Supplementary Table 1 — Brain areas significantly activated in unmasked Ng>Ar contrast (without masking with digit identification task) in addition to the areas found in masked contrast. [file Presentation1.PDF]
